# The cost-effectiveness of neonatal screening for Cystic Fibrosis: an analysis of alternative scenarios using a decision model

**DOI:** 10.1186/1478-7547-3-8

**Published:** 2005-08-09

**Authors:** Neil Simpson, Rob Anderson, Franco Sassi, Alexandra Pitman, Peter Lewis, Karen Tu, Heather Lannin

**Affiliations:** 1 Department of Child Health, Newbridge Hill, Bath, BA1 3QE, UK; 2 Peninsula Technology Assessment Group (PenTAG) & Institute for Health & Social Care Research, Peninsula Medical School, Universities of Exeter and Plymouth, Plymouth, PL6 8BU, UK; 3 Department of Social Policy, The London School of Economics and Political Science, Houghton Street, London, WC2A 2AE, UK; 4 LSE Health and Social Care, The London School of Economics and Political Science, Houghton Street, London, WC2A 2AE, UK; 5 Department of Medical Sciences, University of Bath, Bath, BA2 2 BB, UK; 6 University of Toronto, Canada. Associate Scientist, Institute of Clinical Evaluative Sciences (ICES), G-214, 2075 Bayview Avenue, Toronto, Ontario, M4N 3M5, Canada

## Abstract

**Background:**

The use of neonatal screening for cystic fibrosis is widely debated in the United Kingdom and elsewhere, but the evidence available to inform policy is limited. This paper explores the cost-effectiveness of adding screening for cystic fibrosis to an existing routine neonatal screening programme for congenital hypothyroidism and phenylketonuria, under alternative scenarios and assumptions.

**Methods:**

The study is based on a decision model comparing screening to no screening in terms of a number of outcome measures, including diagnosis of cystic fibrosis, life-time treatment costs, life years and QALYs gained. The setting is a hypothetical UK health region without an existing neonatal screening programme for cystic fibrosis.

**Results:**

Under initial assumptions, neonatal screening (using an immunoreactive trypsin/DNA two stage screening protocol) costs £5,387 per infant diagnosed, or £1.83 per infant screened (1998 costs). Neonatal screening for cystic fibrosis produces an incremental cost-effectiveness of £6,864 per QALY gained, in our base case scenario (an assumed benefit of a 6 month delay in the emergence of symptoms). A difference of 11 months or more in the emergence of symptoms (and mean survival) means neonatal screening is both less costly and produces better outcomes than no screening.

**Conclusion:**

Neonatal screening is expensive as a method of diagnosis. Neonatal screening may be a cost-effective intervention if the hypothesised delays in the onset of symptoms are confirmed. Implementing both antenatal and neonatal screening would undermine potential economic benefits, since a reduction in the birth incidence of cystic fibrosis would reduce the cost-effectiveness of neonatal screening.

## Background

Cystic fibrosis is an inherited disorder associated with considerable morbidity and reduced life expectancy. The UK birth prevalence is about 0.4 per 1,000, or 300 new cases each year [1,2]. A recent report from the UK NHS Health Technology Assessment Programme recommended that antenatal screening for cystic fibrosis should be offered routinely, and that "Health Authorities could consider introducing neonatal screening" [3].

Antenatal screening aims to prevent affected births and neonatal screening aims to improve prognosis by early intervention. Studies of the effectiveness of neonatal screening have measured short-term outcomes or are subject to statistical biases, including selection and lead-time bias, and the use of historical controls [4-8]. Thus the ability of neonatal screening to alter long-term prognosis is not proven, although there is limited circumstantial evidence favouring a benefit [3].

All four existing economic evaluations of neonatal screening for cystic fibrosis are from the USA, and are ten or more years old [9-12]. None used any measure of health effect or performed extensive sensitivity analysis, and only two of the studies compared screening to a do-nothing alternative. Exclusion of the no-screening alternative seems inappropriate, as the type and scale of the benefits of cystic fibrosis screening remain uncertain.

Given the short-comings of these studies, and the lack of full economic evaluations based on UK service and survival data, we aimed to compare the lifetime cost-effectiveness of neonatal screening with no screening, under different possible scenarios for survival with cystic fibrosis. We undertook this from the perspective of a hypothetical UK Health Authority that has an existing routine neonatal screening programme for congenital hypothyroidism and phenylketonuria but not for cystic fibrosis.

## Methods

**Figure 1:**
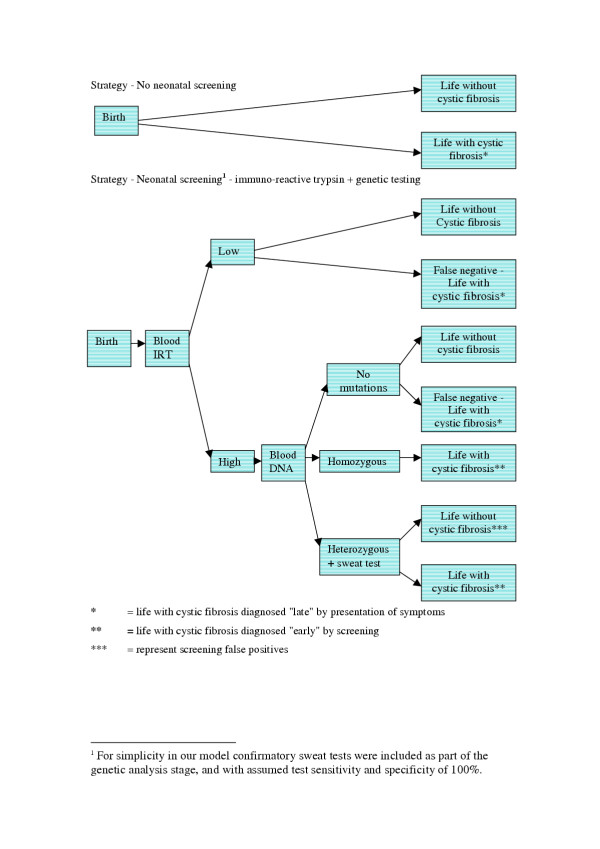
Decision tree for no screening and neonatal screening strategies for cystic fibrosis.

To allow a full exploration of the uncertainty surrounding our cost-effectiveness estimates, they were created using a decision tree, incorporating Markov processes to model lifetime costs and quality of life [13]. We modelled a screening programme using two-stage immunoreactive trypsin combined with genetic testing strategy (Figure 1). This model has replaced two stage immuno-reactive trypsin tests in a number of programmes internationally, and is the commonest protocol used in programmes starting after 1990 [3].

The disease progression was modelled as a Markov process. All cases are 'born' into the pre-symptomatic health-state. Then, each year, there is a given probability of moving into the symptomatic disease state, then into the severe irreversible lung disease-state, and finally death (with the probabilities of moving into the last two states changing over time). These states broadly correspond with the stages of disease described in paediatrics, and also reflect thresholds between different therapy regimes [14].

The model excluded those cases (15% in the base case scenario) diagnosed at or shortly after birth, for example by meconium ileus or family history, since these infants would have received the same prognosis and treatment under both strategies. Under the "no screening" strategy infants would be diagnosed with cystic fibrosis symptomatically (late diagnosis). Under the screening strategy most cystic fibrosis cases would be detected by screening (early diagnosis), with the remainder – the false negatives – experiencing the disease under late diagnosis assumptions.

The putative benefit of early diagnosis through neonatal screening was modelled as a difference in the annual transition probability of remaining pre-symptomatic. In the initial model this probability was 69% for those diagnosed through screening (compared with 59% for those diagnosed symptomatically) resulting in a delay of the emergence of symptoms of 6 months.

The decision analysis model uses three types of data; probability data, cost data, and quality of life estimates for the three health states: these data were obtained from a variety of different sources as referenced in Tables 1, 2 and 3.

**Table 1:** Model parameters, data sources and values used in the model.

**Probabilities**		
Variable	Base case value	Range used in sensitivity analysis

Incidence of cystic fibrosis [2]	0.0004	0.00067 – 0.00029
% diagnosed at birth (MI & family history) [39]	0.15	0.10 – 0.40
IRT test sensitivity [40]	0.9	0.99
IRT test specificity [40]	0.995	0.999
DNA test: % of mutations detected [40]	0.88	0.85 – 0.95
DNA test sensitivity [40]	0.9856	0.9975
DNA test specificity^a^	1.0	
Increased annual transition probability of remaining without symptoms (in early-diagnosed cases)^b^	10%	10 – 40%

^a ^It is assumed that there are no false positives (from the combined DNA and sweat tests), because all test results that are either homozygous or heterozygous for cystic fibrosis mutations, are confirmed using sweat tests.

^b ^Assumed effect of early diagnosis

**Table 2:** Model parameters, data sources and values used in the model.

**Costs (all inflated to reference year 1998)**		
**Variable**	Base case value	Range used in sensitivity analysis

**Costs of screening**		

Additional time to explain test (survey by NS)	£0.40^a ^(2.1 mins)	£0 – £1.44 (0 – 7.6 mins)
Obtaining and transport of blood sample^b^	£0	
IRT test (Bradley DM – pers. comm.)	£0.97	£0.50 – £1.50
DNA test (Bradley DM – pers. comm.)	£79.48	£40.00 – £120.00
Sweat test (Walker S – pers. comm)	£29.40	£15.00 – £45.00
Administration and feedback of results^c^	£0	

**Cost of pre-diagnosis care in unscreened group (audit by NS)**		

Presumed GP visits (mean number of visits)^d^	£14.77 (1.27)	£11.63 – £46.52 (1 – 4)
Outpatient attendances (mean number)^e^	£129.07 (1.47)	£0 – £263.40 (0 – 3)
Inpatient admissions (mean number of admissions and days per admission)^f^	£792.55 (0.87) (3.0 days)	£0 – £1821.96 (0–2)

Costs of treatment per year in health state by age group ^g14^		

Presymptomatic	0–5	£2,950
	6–10	£3,995
	11–15	£4,570
	> 16	£4,275
Symptomatic	0–5	£15,241
	6–10	£15,704
	11–15	£19,247
	>16	£19,291
Severe irreversible symptoms	0–5	£28,722
	6–10	£30,692
	11–15	£37,224
	>16	£37,388

^a ^Whitley Councils for the Health Services Pay Scales – 1/4/98 (Nursing and Midwifery) + 16% on-costs = £11.33/hour

^bc ^Assumed to be a shared cost with existing screening programmes

^d ^The Government's Expenditures Plans 1996–97, Cost/GP consultation = £11.63

^e ^Annual financial returns for NHS Trusts 97/98, Cost//paediatric outpatient attendance = £87.80

^f ^Annual financial returns for NHS Trusts 97/98, Cost//patient-day in paediatric ward) = £303.66

^g ^Hospital staff, overhead and capital costs are included in the average cost per day for inpatient stays and the average cost of outpatient attendance. Drug costs were derived from the monthly index of medical specialities (MIMMS) and do not include individual hospital discount arrangements (1996 costs inflated to 1998).

**Table 3:** Model parameters, data sources and values used in the model.

Utility values of symptom states		
Variable	Base case value	Range used in sensitivity analysis

Asymptomatic – late^a^	0.95	0.90
Asymptomatic – early^b^	0.95	0.90
Symptoms (FEV1 – 60%, range 40%–80%) [17,41]	0.75	0.65 – 0.90
Severe irreversible symptoms (FEV1 – 30%, range 20–40%) [17,41]	0.68	0.58 – 0.78

^a ^utility value to reflect "taking regular medication or staying on a prescribed health diet for health reasons" derived from community surveys.

^b ^arbitrary utility weight to reflect the probable repeated visits to GP with undiagnosed CF

### Probability data

**Figure 2:**
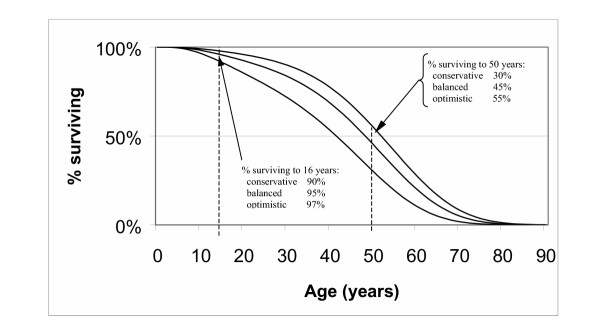
Survival curves under conservative, balanced and optimistic assumptions.

The transition probabilities in the Markov model were estimated to achieve age-specific survival rates and other estimated parameters, in three alternative scenarios; based on conservative, balanced and optimistic assumptions of recent UK age-specific survival data supplied by one of the authors, PL (Figure 2 and Table 3). The annual transition probabilities that best predicted these calibration data were (for the balanced scenario, 'late diagnosis'): from asymptomatic to symptomatic, 0.491 per year (with the remainder all staying asymptomatic); symptomatic to severe irreversible lung disease, 0.0064 increasing exponentially according the accumulated years with symptoms (with hazard rates derived from Dodge et al. 1997); severe irreversible lung disease to death, 0.09 increasing according to the number of years spent in the severe irreversible disease stage. (Excel spreadsheets available on request from the first author, NS). These allowable transitions effectively make the simplifying assumptions that all people with CF ultimately die of CF-related respiratory symptoms, and that all pass through both the symptomatic and severe irreversible lung disease stages before they die.

### Cost data

The following screening costs were the included in the model: counselling time required by midwives to obtain consent for testing, immunoreactive trypsin test, DNA analysis and sweat chloride test. Other costs related to obtaining the blood spot and feedback of results by health visitors, were assumed to be sunk in the existing neonatal screening programmes for phenylketonuria and congenital hypothyroidism.

We assume the addition of cystic fibrosis screening does not increase refusals or insufficient blood samples and that 100% of the neonatal population would be covered by the programme [15]. Time for genetic counselling for carriers identified by the screening programme were excluded from the model.

The pre-diagnosis healthcare costs for children with late diagnosis (no-screening) was estimated via an audit of the clinical notes of 25 children with cystic fibrosis, attaching unit costs to derive the mean cost of pre-diagnosis care (Table 2).

Disease state-specific costs of treatment were derived from the cost of care of 161 patients at a large UK cystic fibrosis unit during 1996 which were based on annual medical costs for patients at different age groups and at different disease stages (Table 2) [14]. Only CF-related health care costs are included, including any costs incurred during additional years of life added by early diagnosis. All cost data was adjusted for inflation at 5% to the reference year of 1998. Future treatment costs in the model were discounted at 6% per year [16].

### Effectiveness data

It has been shown [17,18] that forced expiratory volume in one second (FEV1) is associated with quality of life, as measured by the Quality of Well-Being Scale (a preference-based quality of life instrument used in economic evaluation) [19], as well as with morbidity and mortality [20,21]. These Quality of Well-Being scores remain the only preference-based estimates of health-related quality of life in people with CF. On the basis of these findings, a quality of life value was assigned to each Markov state (Table 3) and multiplied by survival time in the same state to produce quality-adjusted life expectancy. Future QALYs were discounted in the model (2% in base case analysis) [16].

A wide range of one-way, and selected multi-way sensitivity analyses were undertaken. The sensitivity analyses reported here are those that either (a) had a significant impact on the incremental cost-effectiveness ratio, or (b) related to parameters for which reliable published estimates were not available (e.g. QALYs per year of life lived with CF symptoms or severe irreversible symptoms, and the cost of pre-diagnosis care for those diagnosed symptomatically). Microsoft Excel^© ^(version 5.0) spreadsheet software was used to develop the model and conduct the analyses – the models are available on request from the lead author.

## Results

Our base case assumptions gave an estimated cost per diagnosed infant of £5,387 (or £1.83 per infant screened), compared with an estimated cost per case diagnosed clinically of £936. £4,020 (75%) of this cost is due to those components of the screening process that are carried out for every infant screened i.e. the immunoreactive trypsin test, and the explanation of the test by midwives (Table 4).

**Table 4:** Components of the average cost of diagnosis by screening

Cost component	Mean cost of diagnosis per infant screened (£)	Mean cost of diagnosis per infant diagnosed (£)	Cost to average Health Authority^a ^per year (£)
Midwife time explaining test	0.40	1,167	2,380
IRT tests (kits, & overheads)	0.97	2,853	5,820
DNA tests (kits, labour & overheads)	0.42	1,240	2,530
Confirmatory sweat tests	0.01	22	44
Pre-diagnosis care of false negatives	0.04	106	216
Total average cost	1.83	5,387	10,990

^a ^Health Authority with a population of 500,000 and assumed crude birth rate of 12 per 1000 population per year.

**Figure 3:**
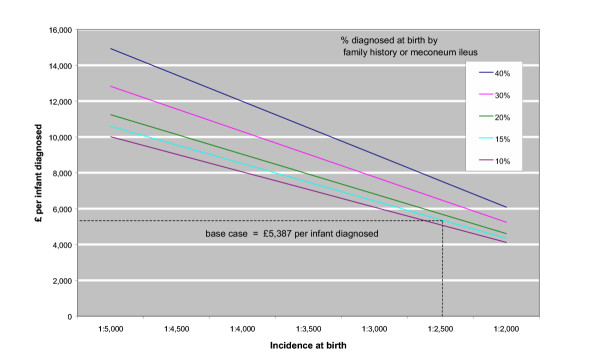
Cost of diagnosis (£) by screening assuming different disease incidence and different proportions diagnosed at birth by other means.

If the explanation to parents of cystic fibrosis screening could be incorporated within existing screening arrangements, the average cost per diagnosed infant would fall by 22% to £4,220. Conversely, if explaining cystic fibrosis screening cannot be incorporated in the existing process, but instead commits midwives to seven and a half minutes more time [22], then the cost per diagnosed infant rises to £8,443. Even with highly optimistic assumptions regarding the specificity and sensitivity of both screening tests the mean cost of diagnosis by screening falls by only a fifth, to £4,351. The cost of diagnosis is more sensitive to the incidence of the disease, and the proportion of cases detected at birth (Figure 3).

**Figure 4:**
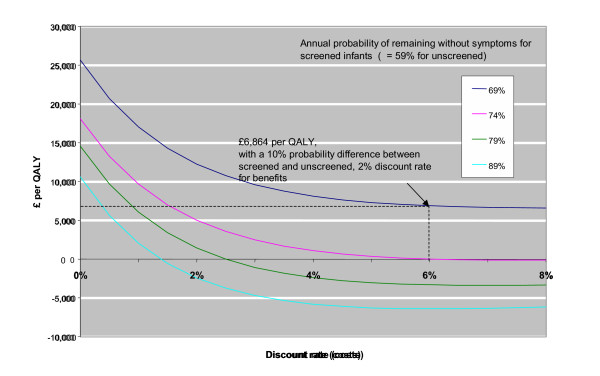
Incremental cost-effectiveness (£/QALY) of neonatal screening for cystic fibrosis compared with no screening, assuming different cost discount rates and assumed effects of preventive therapy.

Neonatal screening for cystic fibrosis produced (under base case assumptions) an average of 0.36 additional QALYs per life with cystic fibrosis at an additional cost of £2,895 (or a cost per QALY gained of £6,864) (Figure 4).

A delay in the emergence of symptoms, or an increase in survival of 11 months or more (i.e. an increase in the probability of remaining without symptoms of 15 percentage points or more), compared to unscreened infants, would produce lower costs and better outcomes than no screening (Figure 4). A wide range of different quality of life estimates for the two disease states produce negligible changes in the cost-effectiveness ratio. This is because in this model the main effect of early diagnosis is to delay the emergence of symptoms, but the progression of disease thereafter is the same under the late and early diagnosis assumptions.

If pre-diagnosis care (of those diagnosed symptomatically) does not involve admission to hospital then the incremental cost-effectiveness ratio falls by 32% to £4,640 per QALY. Under any of the survival models if annual treatment costs are increased by as much as 20% the cost per QALY gained only increases by 11%. Also, as life expectancy increases so does the incremental cost-effectiveness of neonatal screening (Table 5).

**Table 5:** Cost-effectiveness of screening compared to no screening under various survival scenarios

Scenario:	Conservative survival^a^	Balanced survival^a^	Optimistic survival^a^
Model parameter:			
% surviving to age 16	90%	95%	97%
% surviving to age 30	72%	84%	90%
% surviving to age 50	30%	45%	55%
Median survival (years)	41	48	52
Mean survival (years)	39.4	45.8	49.9
Mean years spent:			
before symptoms emerge	1.0	1.0	1.0
with symptoms	32.3	38.7	42.8
in severe irreversible stage	6.1	6.1	6.1
Incremental C/E ratio (£/QALY)	£7,474	£6,864	£6,532

^a ^Under late diagnosis assumptions

## Discussion

As a method of diagnosis neonatal screening is relatively expensive. At £5,387 per diagnosed infant, our estimate of the cost of diagnosis is similar to a previous (1997) estimate of £6,400 [23]. Compared to no screening, neonatal screening for cystic fibrosis (under base case assumptions) produces an incremental cost-effectiveness of £6,864 per QALY gained.

There are a number of key assumptions and potential limitations to this study. Firstly, in relation to the model structure, we have simplified the representation of cystic fibrosis. However our representation of the disease – as three health states within a Markov model – makes best use of available knowledge. Also, we have assumed that the effect of early diagnosis is a delay in the emergence of respiratory symptoms after prophylactic treatment (but, with the subsequent treatment cost and quality of life in those states being the same for screened and unscreened infants). This choice was made in the absence of evidence to suggest other ways of modelling the effect of early diagnosis. Unfortunately, while recent trials or observational studies have shown that early (neonatal) diagnosis and treatment results in improvements in nutritional status, height and weight, and cognitive functioning, evidence about the long-term impact of early diagnosis on lung function (and therefore mortality) remains uncertain [24-28].

The model assumes that cystic fibrosis in infants is a relatively homogeneous condition. However, the spectrum of cases ranges from neonates that are severely affected, to cases who live a normal life undiagnosed until adulthood. It is possible that the more severe but asymptomatic cases would benefit most from early diagnosis. Those with milder forms of the disease would be diagnosed later under the no-screening strategy and would have their age at diagnosis advanced most under screening. The data in existing population-based data sets are insufficient to investigate this issue (PL – author), so this point has been ignored.

Our survival estimates are based on the most recent and reliable age-specific survival data. As yet there is a limited understanding of the interactions of cystic fibrosis with the normal ageing process. Recent clinical experience indicates that potentially life-shortening complications such as diabetes mellitis and liver disease may become more frequent with advancing age, so projections based on experience of younger people may be inaccurate [2,29]. Although it is possible that recent improvements in survival may be confounded by the introduction of neonatal screening in some areas in the 1970s and 1980s, regional variations in mortality do not show this (PL – author). Further, using Quality of Well-Being (QWB) scores as a basis for weighting the quality of extra years survived may not provide a true indicator of relative preferences for being in these different health states. However, it remains the only health-related quality of life instrument that has been used widely in people with cystic fibrosis [30].

Overall, as far as presently available data allow, the model structure and data inputs would satisfy most of the criteria that are recommended in current guidelines for good practice in decision analytic modelling [31]. External validation of the model is more problematic since valid data about either the long-term health effects of screening or the future survival of people with cystic fibrosis does not yet exist. Due to the absence of published data on the distributions underlying the means of most parameters our sensitivity analysis is restricted to one-way and two-way sensitivity analysis. Future modelling of these policy choices should attempt to use data that allow more probabilistic sensitivity analysis to be conducted, but with the proviso that model structure (or methodological) uncertainty can only be explored using traditional 'non-probabilistic' methods.

A number of costs, which may in theory are important have been omitted, either because of the complexity of deriving estimates, or their probable minimal effect on the main findings. The omitted costs are; the potential effects of distress or reassurance related to screening [32], self- and lay-care costs (for example, therapy provided by parents or by the patients themselves), unrelated health care costs and savings resulting from increasing life expectancy (for example, additional years of economically productive life); the costs of genetic counselling (generated by the identification of carriers); and the treatment option of heart-lung transplantation.

The omission of genetic counselling costs was partly because the benefits of such counselling would be difficult to quantify. Existing evidence also shows that, of the small number of carriers that will be identified, only a minority take up counselling and the cost of providing this counselling is small in relation to total screening costs [33,34]. With regard to heart-lung transplantation, this treatment option is currently only available to a relatively small proportion of people with cystic fibrosis. Even if this changes there is no reason to assume that the costs, benefits or availability would be different for those diagnosed at birth by screening and those diagnosed later symptomatically [35-39].

With regard to the generalisability of the findings, this study has most relevance to the UK context: it employs cost estimates based on NHS care and the survival estimates are derived from the UK National Cystic Fibrosis Survey [1]. Although comparisons with other studies are difficult; the costs of care at the Leeds unit (average annual cost of £10,567 [14]) from which our data are derived were comparable with another UK study [40].

Although a number of alternative screening protocols are being used in the UK and world-wide, they all employ an IRT test as the initial screening stage for all neonates. Our analysis shows that it is the cost of this initial stage, carried out on all infants, which most affects the cost-effectiveness ratio, and also that differences in the performance of the screening protocol produce only minor changes. Therefore, it is unlikely that substantially different results would be obtained with alternative protocols.

We have shown that, in the absence of antenatal screening, neonatal screening costs of £6,864 per QALY gained, based on an assumed benefit of six months average delay in the onset of symptoms, and that it would be less costly and more beneficial if the benefit were shown to be 11 months or more. As further evidence becomes available it will be clearer if this threshold can be realised. The model used here could be adapted to reflect new effectiveness data, associations between genotype and phenotype, new treatments for cystic fibrosis as they become available; and local information concerning populations or services.

Comparisons of cost per affected pregnancy identified by antenatal screening with averted treatment costs are generally favourable [3]. However, these studies give no value to a life lived with cystic fibrosis. They also assume around 90% uptake of prenatal diagnosis and effectively universal termination of affected pregnancies. This contrasts with surveys of affected individuals and close family members which suggest that only about half find termination of an affected pregnancy acceptable [41-43]. Changes in public attitudes about prenatal diagnosis and termination might further affect the economic value of antenatal screening.

In the UK policy context Murray *et al*. recommended both that antenatal screening should be offered routinely and that health authorities 'could consider' introducing neonatal screening. They also suggest that routine antenatal screening would reduce the birth prevalence of cystic fibrosis by between 43% and 49% [3]. According to our analysis, by halving the birth prevalence of the disease, the cost per QALY gained for neonatal screening would increase to £19,543. With this lower prevalence at birth, early diagnosis would have to delay the emergence of symptoms on average by 20 months or more for neonatal screening to be less costly and more beneficial.

In 1998 in the UK, up to a quarter of the population were covered by six regional programmes of neonatal screening for cystic fibrosis and we are aware of only one scheme which provides routine antenatal screening (in Edinburgh) [3]. Any economic evaluation of antenatal screening compared with neonatal screening is impossible as the strategies have such different aims. Fundamentally, the benefits are not comparable: in antenatal screening the aim is to allow reproductive choice, including the option to terminate affected pregnancies, whereas neonatal screening primarily aims to improve the length and quality of life of sufferers. Economic evaluations of antenatal screening have therefore tended to give no value to a life with cystic fibrosis, and instead attribute financial savings to lives with cystic fibrosis avoided.

In conclusion, according to the scenarios explored here, as long as the birth incidence of cystic fibrosis remains stable, there is no reason for existing neonatal screening programmes to be discontinued on cost-effectiveness grounds. Although UK Health Authorities wanting to introduce neonatal screening may want to see more reliable evidence of the health benefits of early diagnosis before making a decision, this evidence is now beginning to emerge, especially from the Wisconsin trial [44]. It has prompted the (UK) NHS National Screening Committee to implement a national neonatal screening programme for England (Scotland introduced theirs in 2003). As this programme is rolled out to different regions from April 2005 (see ) more reliable data on how early diagnosis alters lung function and long-term survival will become available, and could be used to update this cost-effectiveness analysis.

## Competing interests

The author(s) declare that they have no competing interests.

## Authors' contributions

NS, RA, FS, AP, KT, HS all contributed to conception, design of study and editing, RA developed spreadsheet model, NS, RA and FS undertook analysis and wrote the paper, and PL contributed to analysis and editing.

## References

[B1] Dodge JA, Morison S, Lewis PA, Coles E, Geddes D, Russel G (1993). Cystic Fibrosis in the United Kingdom, 1968–1988: incidence, population and survival. Pediatr & Perinat Epidemiol.

[B2] Dodge JA, Morison S, Lewis PA, Coles E, Geddes D, Russel G (1997). Incidence, population and survival of cystic fibrosis in the UK, 1968–95. Arch Dis Child.

[B3] Murray J, Cuckle H, Taylor G, Littlewood J, Hewison J (1999). Screening for cystic fibrosis. Health Technology Assessment.

[B4] Farrell PM, Kosorok MR, Laxova A, Guanghong S, Koscik R, Bruns T (1997). Nutritional benefits of neonatal screening for cystic fibrosis. N Engl J Med.

[B5] Chatfield S, Owen G, Ryley H, Williams J, Alfaham M, Goodchild M (1991). Neonatal screening for cystic fibrosis in Wales and the West Midlands: clinical assessment after five years of screening. Arch Dis Child.

[B6] Kuzemko JA (1986). Neonatal screening for cystic fibrosis. Lancet.

[B7] Dankert-Roelse J, Meeman GJ, Martijin A, Kate LP, Knol K (1989). Survival and clinical outcome in patients with cystic fibrosis, with or without neonatal screening. J Pediatr.

[B8] Waters D, Wilken B, Irwig L, Van Asperen P, Mellis C, Simpson J (1999). Clinical outcomes of newborn screening for cystic fibrosis. Arch Dis Child Fetal Neonatal Ed.

[B9] Gregg RG, Wilfond BS, Farrell P, Laxova A, Hassemer D, Mischler E (1993). Application of DNA analysis in a population-screening program for neonatal diagnosis of cystic fibrosis (CF): comparison of screening protocols. American Journal of Human Genetics.

[B10] Dauphinais RM (1992). A cost-analysis of blood spot screening of newborns for cystic fibrosis. J Clin Immunoassay.

[B11] Farrell PM, Mischler EH (1992). Newborn screening for cystic fibrosis. Advances in Pediatrics.

[B12] Pauly MV (1983). The Economics of Cystic Fibrosis. Boston, John Wright, PSG.

[B13] Sonnenberg F, Beck R (1993). Markov Models in medical decision making: a practical guide. Medical Decision Making.

[B14] Littlewood JM, Cross E (1999). Present day treatment of cystic fibrosis: its content and cost. Clinical Economics in Gastroenterology.

[B15] Simpson N, Randall R, Lenton S, Walker S (1997). Audit of neonatal screening programme for phenylketonuria and congenital hypothyroidism. Arch Dis Child.

[B16] Torgerson DJ, Raftery J (1999). Economics notes – Discounting. BMJ.

[B17] Orenstein D, Nixon PA, Ross EA, Kaplan RM (1989). The quality of well-being in cystic fibrosis. Chest.

[B18] Orenstein D, Pattishall EN, Nixon PA, Ross EA, Kaplan RM (1990). Quality of well-being before and after antibiotic treatment of pulmonary exacerbation on patients with cystic fibrosis. Chest.

[B19] Bowling A (1991). Measuring Health – a review of Quality of Life measurement scales. Milton Keynes.

[B20] Congleton J, Hodson M, Duncan-Skingle F (1996). Quality of life in adults with cystic fibrosis. Thorax.

[B21] Corey M, Farewell V (1996). Determinants of mortality from cystic fibrosis in Canada, 1970–1989. Am J Epidemiol.

[B22] Simpson N (1999). Audit of time for midwives to explain cystic fibrosis screening. Unpublished.

[B23] Pollitt R, Green A, McCabe C, Booth A, Cooper N, Leonard J (1997). Neonatal screening for inborn errors of metabolism: cost, yield and outcome (Review). Health Technology Assessment.

[B24] Lai HJ, Cheng Y, Cho H, Kosorok MR, Farrel P (2004). Association between initial disease presentation, lung disease outcomes, and survival in patients with cystic fibrosis. American J Epidemiol.

[B25] Koscik RL, Farrel PM, Kosorok MR, Zaremba KM, Laxova A, Lai HC (2004). Cognitive function of children with cystic fibrosis: deleterious effect of early malnutrition. Pediatrics.

[B26] Assael BM, Castellani C, Ocampo MB, Iansa P, Callegaro A, Valsecchi MG (2002). Epidemiology and survival analysis of cystic fibrosis in an area of intense neonatal screening over 30 years. American Journal of Epidemiology.

[B27] Siret D, Bretaudeau G, Branger B, Dabadie A, Dagorne M, David V, de Braekeleer M (2003). Comparing the clinical evolution of cystic fibrosis screened neonatally to that of cystic fibrosis diagnosed from clinical symptoms: a 10-year retrospective study in a French region (Brittany). Pediatric Pulmonology.

[B28] Wang SS, FitzSimmons SC, O'Leary LA, Rock MJ, Gwinn ML, Khoury MJ (2001). Early diagnosis of cystic fibrosis in the new-born period and risk of Pseudomonas aeruginosa acquisition in the first 10 years of life: a registry-based longitudinal study. Pediatrics.

[B29] Lewis P, Morison S, Dodge J, Geddes D, Coles E, Russell G (1999). Survival estimates for adults with cystic fibrosis born in the United Kingdom between 1947 and 1967. Thorax.

[B30] Kotwicki RJ, Condra L, Vereulen L, Wolf T, Douglas J, Farrel PM (2001). Assessing the quality of life in children with cystic fibrosis. Western Medical Journal.

[B31] Weinstein M, O'Brien B, Hornberger J, Jackson J, Johannesson M, McCabe C (2003). Principles of good practice for decision analytic modeling in health-care evaluation: Report of the ISPOR Task Force on Good Research Practices – Modelling Studies. Value in Health.

[B32] Sassi F, McKee M, Roberts J (1997). Economic evaluation of diagnostic technology: methodological challenges and viable solutions. Int J Technolol assessment in Health Care.

[B33] Shannon N, Evans S, Pollitt R, Quarrell O (1997). Follow up of Delta F508 detected as a result of neonatal screening in the Trent Region. J Med Genet.

[B34] Ranieri E, Lewis B, Morris C, Wilcken B, Dodge JBD, Widdicombe JH (1996). Neonatal screening using combined biochemical and DNA based techniques. Cystic Fibrosis – current topics.

[B35] Wiebe K, Wahlers T, Harringer W, vd Hardt H, Fabel H, Haverich A (1998). Lung transplantation for cystic fibrosis – a single centre experience over 8 years. European Journal of Cardio-Thoracic Surgery.

[B36] Coueitil J, Soubrane O, Houssin D, Dousset B, Chevalier P (1997). Combined heart-lung-liver, double lung-liver, and isolated liver transplantation for cystic fibrosis in children. Transplant International.

[B37] Whitehead B, Helms P, Goodwin M, Martin I, Scott J, Smyth R (1991). Heart-lung transplantation for cystic fibrosis. 2: Outcome. Archives of Disease in Childhood.

[B38] Balfour-Lynn I, Martin I, Whitehead B, Rees P, Elliot M, de Leval M (1997). Heart-lung transplantation for patients under 10 with cystic fibrosis. Archives of Disease in Childhood.

[B39] Busschbach J, Horikx P, van den Bosch J, Brutal de la Riviere A, de Charro F (1994). Measuring the quality of life before and after bilateral lung transplantation in patients with cystic fibrosis. Chest.

[B40] Robson M, Abbott J, Webb K, Dodd M, Walsworth-Bell J (1992). A cost description of an adult cystic fibrosis unit and cost analysis of different categories of patients. Thorax.

[B41] Watson EK, Williamson R, Chapple J (1991). Attitudes to carrier screening for cystic fibrosis: a survey of health care professionals, relatives of sufferers and other members of the public. Br J Gen Pract.

[B42] Watson EK, Marchant J, Bush A, Williamson B (1992). Attitudes towards prenatal diagnosis and carrier screening for cystic fibrosis among the parents of patients in a paediatric cystic fibrosis clinic. J Med Genet.

[B43] Conway SP, Allenby K, Pond MN (1994). Patient and parental attitudes toward genetic screening and its implications at an adult cystic fibrosis clinic. Clin Gen.

[B44] Grosse SD, Boyle CA, Botkin JR, Comeau AM, Kharrazi M, Rosefeld M (2004). Newborn screening for cystic fibrosis: evaluation of benefits and risks and recommendations for state newborn screening programs. Morbidity & mortality weekly report.

